# Construction of thyroid cancer classification and iodine metabolism related diagnostic model using thyroid differentiation score and bioinformation

**DOI:** 10.1097/MD.0000000000039464

**Published:** 2024-09-06

**Authors:** Qiu-ying Zhang, Yan Wang, Qiang Zhang, Da-wei Huo, Yue Li, De Gao, Pan-pan Wang, Hai-chao Yan

**Affiliations:** a Surgical Oncology, The Fifth People’s Hospital of Ningxia, Shizuishan, China; b Department of Nephrology, Ningxia Medical University Generai Hospital, Yinchuan, China; c Department of Chinese Medicine, The Fifth People’s Hospital of Ningxia, Shizuishan, China; d Department of Thyroid Surgery, The Second Affiliated Hospital, Zhejiang University School of Medicine, Hangzhou, China.

**Keywords:** diagnostic model, prognosis, thyroid cancer, thyroid differentiation score

## Abstract

To more accurately diagnose and treat patients with different subtypes of thyroid cancer, we constructed a diagnostic model related to the iodine metabolism of THCA subtypes. THCA expression profiles, corresponding clinicopathological information, and single-cell RNA-seq were downloaded from TCGA and GEO databases. Genes related to thyroid differentiation score were obtained by GSVA. Through logistic analyses, the diagnostic model was finally constructed. DCA curve, ROC curve, machine learning, and K-M analysis were used to verify the accuracy of the model. qRT-PCR was used to verify the expression of hub genes in vitro. There were 104 crossover genes between different TDS and THCA subtypes. Finally, 5 genes (ABAT, CHEK1, GPX3, NME5, and PRKCQ) that could independently predict the TDS subpopulation were obtained, and a diagnostic model was constructed. ROC, DCA, and RCS curves exhibited that the model has accurate prediction ability. K-M and subgroup analysis results showed that low model scores were strongly associated with poor PFI in THCA patients. The model score was significantly negatively correlated with T cell follicular helper. In addition, the diagnostic model was significantly negatively correlated with immune scores. Finally, the results of qRT-PCR corresponded with bioinformatics results. This diagnostic model has good diagnostic and prognostic value for THCA patients, and can be used as an independent prognostic indicator for THCA patients.

## 1. Introduction

Thyroid cancer (THCA) is a common malignant endocrine tumor, and its incidence rate shows an increasing trend year by year.^[1]^ The areas with high THCA incidence rates are mainly located in the northern United States, Australia, East Asia, and southern Europe. In China, the incidence rate of THCA is also increasing, and it has become one of the top ten cancers threatening the health of Chinese residents.^[2]^ The incidence rate of THCA is related to various factors including obesity, radiation factors, iodine intake, gene mutation, gender, and family history.^[3,4]^

It is well known that the differentiation status of tumor cells is closely related to the malignancy grade of tumors, and the lower the degree of differentiation, the greater the potential malignancy of the tumor.^[5]^ In particular, the differentiation of THCA cells plays a central role in THCA development.^[6]^ THCA is classified into differentiated thyroid cancer (DTC), anaplastic thyroid carcinoma (ATC), and poorly differentiated thyroid cancer (PDTC) according to its originating cells.^[7]^ Papillary thyroid carcinoma (PTC) belongs to the highly differentiated type, accounting for more than 80% of THCA patients, with a good clinical prognosis and a 10-year disease specific survival rate of over 90%; ATC belongs to the undifferentiated type, accounting for nearly 50% of thyroid-related deaths, with stem cell-like characteristics, high proliferation potential, and drug resistance, with a median survival time of only 6 months.^[8]^ Histologically, PTC develops into ATC through dedifferentiation, which is a biological process that can induce tumor cells to transition from a highly differentiated state to a poorly differentiated state.^[9]^ Compared with DTC, ATC may lack some biological features or functions, such as thyroglobulin synthesis, TSH dependence, and iodine uptake.^[10]^ In addition, iodine intake plays an essential role in the development of thyroid diseases.^[11]^ Radioactive iodine (RAI) therapy takes a vital role in the treatment of THCA, and its therapeutic effect on differentiated THCA is good, but its therapeutic effect on poorly differentiated THCA is worrying.^[12,13]^ Some studies have shown that nearly 5% of patients underwent cell dedifferentiation, accompanied by more aggressive growth, metastatic spread, and loss of iodine uptake ability, making THCA resistant to conventional treatment and RAI therapy during the progression of THCA.^[5]^ Other studies have shown that RAI treatment is closely related not only to thyroid follicular absorption but also to the ability to concentrate iodized compounds.^[14]^ Research has found that thyroid differentiation score (TDS), which is calculated by DIO1, DIO2, DUOX1, DUOX2, FOXE1, GLIS3, PAX8, NKX2-1, SLC26A4, SLC5A5, SLC5A8, TG, TPO, TSHR, THRA, and THRB,^[15]^ is a comprehensive index related to the expression and function of iodine metabolism related genes.^[6,15]^ Many studies have identified predictive factors for malignant tumors by conducting gene analysis and screening biomarkers, but their sensitivity in predicting THCA differentiation is not favorable.^[16]^ Therefore, we constructed a diagnostic model that was both related to THCA subtype and iodine metabolism using TDS, aiming to provide new methods for the diagnosis and treatment of THCA.

In this study, we constructed a multi-gene diagnostic model related to THCA subtypes and TDS using bioinformatics and machine learning algorithms based on public databases. Besides, we also further explored the value of this model in the diagnosis, prognosis, and immunotherapy of THCA patients. In summary, our results indicated that the diagnostic model may play an important role in the diagnosis, prognosis, and treatment of THCA patients.

## 2. Methods

### 2.1. Data collection

We downloaded the THCA expression and clinical pathological information from The Cancer Genome Atlas (TCGA) database (https://tcga-data.nci.nih.gov/tcga/), including gender, age, pathological stage, etc. We excluded samples with incomplete expression and clinical information, as well as normal samples. Ultimately 489 samples were included for analysis. Subsequently, gene expression data and relevant clinical information from the GSE33630 dataset (including 17 normal, 49 PTC, and 11 ATC samples) and the GSE29265 dataset (including 20 normal, 20 PTC, and 9 ATC samples), collected on the GPL570 platform, were downloaded from the Gene Expression Omnibus (GEO) database (https://www.ncbi.nlm.nih.gov/geo/). Single-cell sequencing data were obtained from the GSE193581 dataset, which included 2 NT samples, 2 PTC samples, and 2 ATC samples.

### 2.2. Acquisition of TDS

We calculated the TDS of each sample based on the TCGA-THCA mRNA expression levels of 16 thyroid functional genes. The obtained TDS can quantify the relationship between thyroid differentiation and various genetic or epigenetic events. TDS = Mean of Log2 (Fold Change) across 16 genes. The Log2-normalized RSEM values were first centered at the median across samples, yielding Log2 (Fold Change), and then summed across the 16 genes for each sample: TDS = Mean of Log2 (Fold Change) across 16 genes.

### 2.3. Gene set variation analysis

Gene set variation analysis (GSVA) is a non-parametric and unsupervised analysis method that can evaluate the potential changes in pathway activity in each sample.^[17,18]^ We used the GSVA R package to calculate the enrichment scores of 186 Kyoto Encyclopedia of Genes and Genomes signaling pathways for each sample in the TCGA-THCA dataset and finally obtained an enrichment score matrix. Besides, the TDS was also calculated using the GSVA R package after the related genes were determined in a previous study.^[15]^

### 2.4. The determination of TDS-related genes

Firstly, we calculated TDS using GSVA and analyzed the correlation between TDS and 186 signal path enrichment scores using the corrr package in R. The screening criteria for TDS-related signaling pathways were |Cor| > 0.5 and *P* < .05. Secondly, we used the gene set enrichment analysis database (https://www.gsea-msigdb.org/) to obtain genes related to signaling pathways.

### 2.5. Acquisition of THCA subtype-related genes

We used the limma package in R language to identify differentially expressed genes (DEGs) between PTC patient-NT samples and PTC patient-ATC samples in the GSE33630 dataset. The threshold we set was |log2(Fold change)| > 2.0 and *P* < .05.

### 2.6. Acquisition of intersection genes

We used the VennDiagram package in R software to obtain the intersection of TDS-related genes and THCA subtype-related genes as TDS-THCA subtype-related genes.

### 2.7. Construction of a diagnostic model

First, we further screened TDS-THCA subtype-related genes using the least absolute shrinkage and selection operator (LASSO) analysis in a machine learning algorithm. Secondly, we used the corrplot package to calculate the correlation between genes and excluded those with Cor > 0.6. In addition, we screened out important TDS-THCA subtype-related genes using the results of difference analysis in the GSE29265 dataset. Then, we divided the sample into high-expression and low-expression groups based on the median TDS score, and performed logistic regression analyses for important TDS-THCA subtype-related genes. Based on multivariate logistic regression analysis results, we constructed a diagnostic model related to TDS group.

### 2.8. Diagnostic analysis of the diagnostic model

Firstly, we used the plotRCS package in R to perform restricted cubic spline (RCS) analysis and verified whether the diagnostic model was nonlinearly related to TDS group. Next, we respectively used the ggDCA and pROC packages in R based on TCGA data to perform decision curve analysis (DCA) and receiver operating characteristic (ROC) analysis, which could evaluate the diagnostic value of the model for TDS group. Additionally, we assessed the predictive efficacy of the diagnostic model for THCA subgroup using multiclass ROC analysis based on the GSE33630 and GSE29265 datasets. Finally, we divided the TCGA-THCA dataset into a 4:1 ratio of training set and validation set, and used multiple machine learning algorithms to further verify the effectiveness of diagnostic model in distinguishing normal and THCA samples. Machine learning algorithms included XGBoost, logistic, RandomForest, DecisionTree, KNN, and SVM.

### 2.9. Prognostic analysis of the diagnostic model

We firstly used Kaplan–Meier (K-M) curves to present the relationship between the diagnostic model and prognosis in THCA patients. In addition, we performed subgroup analysis based on gender, age, pathological stage, and histological type. Besides, we used the forestplot package in R to draw forest plots. Finally, univariate and multivariate COX analyses were used to confirm the independent prognostic value of the diagnostic model.

### 2.10. Immune infiltration analysis

We used the CIBERSORTx online platform (https://cibersortx.stanford.edu/) to calculate the scores of 22 immune-infiltrating cells in each sample from the TCGA-THCA, GSE33630, and GSE29265 datasets. In addition, we utilized the ESTIMATE algorithm to calculate the scores of stromata, immune, and ESTIMATE in tumor samples. Finally, we explored the relationship between the diagnostic model and two indicators that can be assessed immune therapy, namely tumor mutational burden (TMB) and microsatellite instability (MSI). Pearson coefficient test was used to examine the correlation between the diagnostic model and the scores of 22 immune cells, stromata, immune, and ESTIMATE.

### 2.11. Single-cell RNA-seq (scRNA-seq) analysis

Firstly, we use the Seurat package of R software to integrate the scRNA-seq data. Secondly, in order to remove low-quality scRNA-seq data, we used three screening criteria as follows: the removal of cells with a total number of genes greater than 200, the elimination of cells with counts less than 1000 genes or more than 20,000 genes, and the elimination of cells with more than 5% mitochondrial genes. The “NormalizeData” function was then used to normalize the data, and the “FindVariableFeatures” function was used to identify 1500 highly variable genes. Further, we use “RunPCA” function for principal component analysis. Subsequently, we used JackStraw analysis to identify significant PCs. We then use the “FindNeighbors” and “FindClusters” functions for cell clustering analysis. Then, we use “RunTSNE” function for T-distributed random neighborhood embedding. After that, we used the Wilcoxon-Mann-Whitney test to calculate the expression difference of each gene in the model in different samples. Finally, we use the SingleR package in R software for cell annotation.

### 2.12. RNA extraction and qRT-PCR

Total RNA was isolated from tissues and cells using TRIzol reagent (Invitrogen, https://www.thermofisher.cn/cn/zh/home/brands/invitrogen.html.html) according to the manufacturer’s protocol. Using reverse transcription kit (Takara, Japan, https://www.takarabiomed.com.cn/DownLoad.aspx?m=20141215094609200088), we reversed transcribed RNA into cDNA. GAPDH was used as an internal control. The expression of each gene was normalized to that of the internal control and quantified using the 2−ΔΔCT method.^[19]^ The primers used were listed as follows: ABAT forward, 5′-AAGAGAGCCGAGGCAATTACC-3′ and reverse, 5′-GCTCGCATTTTGAGGCTGTTG-3′; and CHEK1 forward, 5′-ATATGAAGCGTGCCGTAGACT-3′ and reverse, 5′-TGCCTATGTCTGGCTCTATTCTG-3′; and GPX3 forward, 5′-AGAGCCGGGGACAAGAGAA-3′ and reverse, 5′-ATTTGCCAGCATACTGCTTGA-3′; and NME5 forward, 5′-CGGATTCACCATTGTTCAGAGA-3′ and reverse, 5′-CATGTAAGCTGTTAAGTTGGGGA-3′; and PRKCQ forward, 5′-ATGTCGCCATTTCTTCGGATT-3′ and reverse, 5′-ACATACTCTTTGACGAGCACAG-3′.

### 2.13. Statistical analysis

We used R (Version 3.14.3) for bioinformatics analysis and plotting. Python (Version 3.12.2) was used for machine learning algorithm validation. GraphPad Prism 8.0 and SPSS 25.0 software were used for statistical analysis. In addition, when analyzing the relationship between clinical features and the diagnostic score model, we processed the data using K-S goodness of fit tests. Data conforming to normal distribution were expressed as mean ± standard error, and group comparisons were made using Student *t* test or Analysis of variance. Data that did not conform to the normal distribution were represented by quartiles (Q1–Q4), and the differences between groups were analyzed by rank sum test. *P* < .05 was considered statistically significant.

## 3. Results

### 3.1. The genes related to TDS

**Figure 1.**
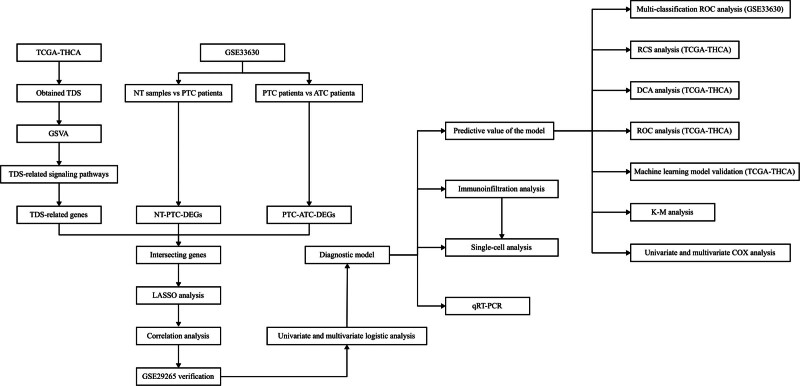
The entire analytical process of the study.

Figure 1 displays the process of this research. We first obtained 27 signaling pathways significantly related to TDS to obtain TDS-related genes according to the screening criteria. Secondly, we obtained 996 genes related to these 27 signaling pathways through the gene set enrichment analysis database (Table 1).

**Table 1:** The correlation between TDS and KEGG signal pathways.

Signaling pathway name	*P* value	Cor
BUTANOATE_METABOLISM	<.001	0.66
PROXIMAL_TUBULE_BICARBONATE_RECLAMATION	<.001	0.66
TERPENOID_BACKBONE_BIOSYNTHESIS	<.001	0.65
GLYCINE_SERINE_AND_THREONINE_METABOLISM	<.001	0.65
GLYCEROLIPID_METABOLISM	<.001	0.65
LIMONENE_AND_PINENE_DEGRADATION	<.001	0.62
TYPE_II_DIABETES_MELLITUS	<.001	0.62
VALINE_LEUCINE_AND_ISOLEUCINE_DEGRADATION	<.001	0.62
LYSINE_DEGRADATION	<.001	0.61
BETA_ALANINE_METABOLISM	<.001	0.61
PROPANOATE_METABOLISM	<.001	0.60
FATTY_ACID_METABOLISM	<.001	0.57
STARCH_AND_SUCROSE_METABOLISM	<.001	0.54
ADIPOCYTOKINE_SIGNALING_PATHWAY	<.001	0.53
ASCORBATE_AND_ALDARATE_METABOLISM	<.001	0.52
PYRUVATE_METABOLISM	<.001	0.52
PROTEIN_EXPORT	<.001	0.51
GLYCOSPHINGOLIPID_BIOSYNTHESIS_LACTO_AND_NEOLACTO_SERIES	<.001	−0.50
PYRIMIDINE_METABOLISM	<.001	−0.50
ASTHMA	<.001	−0.51
CELL_ADHESION_MOLECULES_CAMS	<.001	−0.52
DNA_REPLICATION	<.001	−0.53
NOD_LIKE_RECEPTOR_SIGNALING_PATHWAY	<.001	−0.54
GLYCOSAMINOGLYCAN_DEGRADATION	<.001	−0.54
CELL_CYCLE	<.001	−0.55
ARACHIDONIC_ACID_METABOLISM	<.001	−0.57
P53_SIGNALING_PATHWAY	<.001	−0.62

KEGG = Kyoto Encyclopedia of Genes and Genomes, TDS = thyroid differentiation score.

### 3.2. The genes related to TDS-THCA subtype

**Figure 2.**
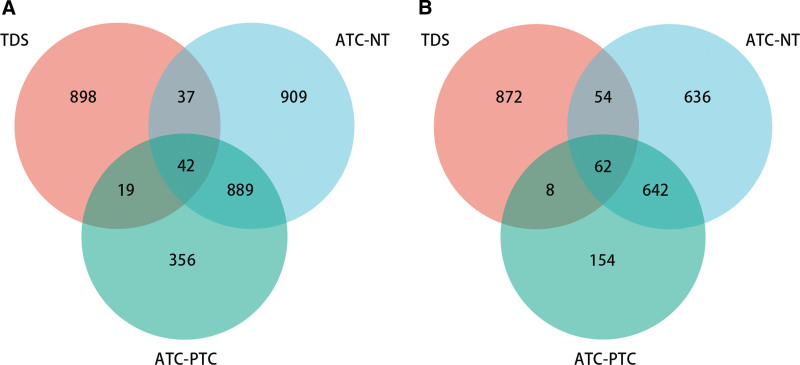
Venn diagram shows the number of overlapping genes of TDS-related genes, ATC-NT and ATC-PTC differential genes. (A) Venn diagram of TDS-related genes, ATC-NT down-regulated genes and ATC-PTC down-regulated genes. (B) Venn diagram of TDS-related genes, ATC-NT upregulated genes and ATC-PTC upregulated genes. ATC = anaplastic thyroid carcinoma, NT = normal thyroid sample, PTC = papillary thyroid carcinoma, TDS = thyroid differentiation score.

To obtain DEGs associated with THCA subtype, we performed differential gene expression analysis between NT samples-PTC patients and PTC patients-ATC patients. The results showed that there were 1877 significantly downregulated genes and 1394 significantly upregulated genes in NT samples-PTC patients; and 1306 significantly downregulated genes and 866 significantly upregulated genes in PTC patients-ATC patients (Fig. 2A and B). The results of Venn analysis exhibited that there were 42 common significantly downregulated genes and 62 common significantly upregulated genes between TDS-THCA subtypes, and a total of 104 DEGs were obtained.

### 3.3. Construction of a diagnostic model

**Figure 3.**
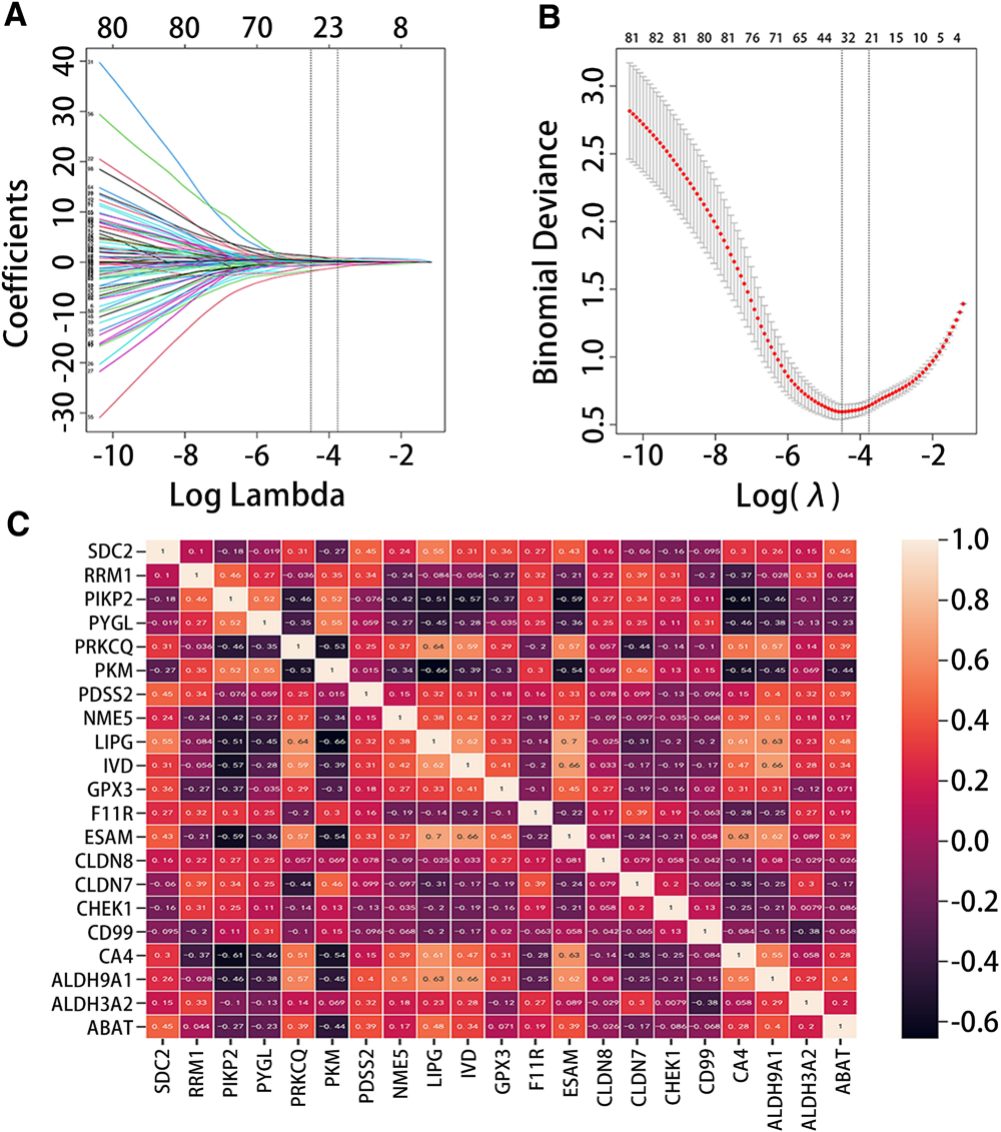
Screening the key genes related to TDS-THCA subtyping. (A-B) LASSO coefficient path diagram and LASSO regression analysis cross-validation curve. (C) heat map of gene correlation. LASSO = least absolute shrinkage and selection operator, TDS = thyroid differentiation score, THCA = thyroid cancer.

**Figure 4.**
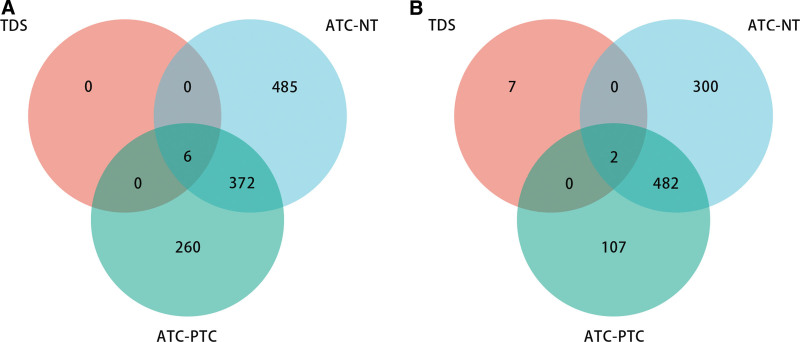
Venn diagram showed the number of overlapping genes related to TDS-THCA subtyping, ATC-NT, and ATC-PTC differential genes. (A) Venn diagram of TDS-THCA subtyping-related genes, ATC-NT down-regulated genes, and ATC-PTC down-regulated genes. (B) Venn diagram of TDS-THCA subtyping-related genes, ATC-NT upregulated genes and ATC-PTC upregulated genes. ATC = anaplastic thyroid carcinoma, NT = normal thyroid sample, PTC = papillary thyroid carcinoma, TDS = thyroid differentiation score, THCA = thyroid cancer.

The LASSO analysis showed that there were 21 genes with non-zero regression coefficients in the TDS-THCA subtype-related gene set (Fig. 3A and B). The correlation analysis results presented that there were 6 strongly correlated genes, and we ultimately retained 15 TDS-THCA subtype-related genes (6 up-regulated genes and 9 down-regulated genes) after the highly correlated genes were removed (Cor > 0.6) (Fig. 3C). Figure 4A and B results exhibited that there were eight DEGs among TDS-THCA subtype-related genes, ATC-NT-related differential genes, and ATC-PTC-related differential genes in the GSE29265 dataset, of which six genes were significantly down-regulated and two genes were significantly up-regulated (Table 2) (*P* < .05). Univariate logistic regression analysis showed that 7 genes were significantly related to high or low TDS scores (Table 3). Multivariate logistic regression analysis identified 5 genes, which could independently predict high or low TDS scores, and constructed a diagnostic model: Model score = 0.649*ABAT-2.338*CHEK1 + GPX3 + 1.516*NME5 + 2.243*PRKCQ-18.976. In the diagnostic model, the expressions of ABAT, GPX3, NME5, and PRKCQ were significantly downregulated, while the expression of CHEK1 was significantly upregulated (Table 2).

**Table 2:** Overlapping genes of TDS-THCA typing-related genes and the results of difference analysis based on the GSE29265 data set.

Gene ID	Gene name	Log2FC	*P* value
ABAT	4-aminobutyrate aminotransferase	−1.670	<.001
CLDN8	Claudin-8	−3.778	<.001
GPX3	Glutathione Peroxidase 3	−2.972	<.001
NME5	NME/NM23 Family Member 5	−1.690	<.001
PRKCQ	Protein Kinase C Theta	−2.100	<.001
SDC2	Syndecan 2	−1.920	<.001
CHEK1	Checkpoint Kinase 1	1.537	<.001
PYGL	Glycogen Phosphorylase L	1.781	<.001

TDS = thyroid differentiation score, THCA = thyroid cancer.

**Table 3:** Correlation between TDS-THCA typing-related genes and TDS group.

Characteristics	Total (N)	Univariate analysis		Multivariate analysis	
		Odd ratio (OR)	*P* value	Odd ratio (OR)	*P* value
CLDN8	385	0.907 (0.746–1.102)	.326		
ABAT	385	0.406 (0.302–0.545)	<.001	1.878 (1.188–2.971)	.007
CHEK1	385	9.078 (3.637–22.659)	<.001	0.097 (0.026–0.364)	.001
GPX3	385	0.323 (0.246–0.425)	<.001	2.666 (1.882–3.776)	<.001
NME5	385	0.082 (0.041–0.166)	<.001	4.566 (1.781–11.703)	.002
PRKCQ	385	0.064 (0.036–0.113)	<.001	9.647 (4.908–18.963)	<.001
PYGL	385	2.274 (1.589–3.254)	<.001	1.063 (0.627–1.803)	.820
SDC2	385	0.354 (0.253–0.495)	<.001	1.079 (0.667–1.746)	.757

TDS = thyroid differentiation score, THCA = thyroid cancer.

### 3.4. Value of diagnostic model in classification of patients with THCA

**Figure 5.**
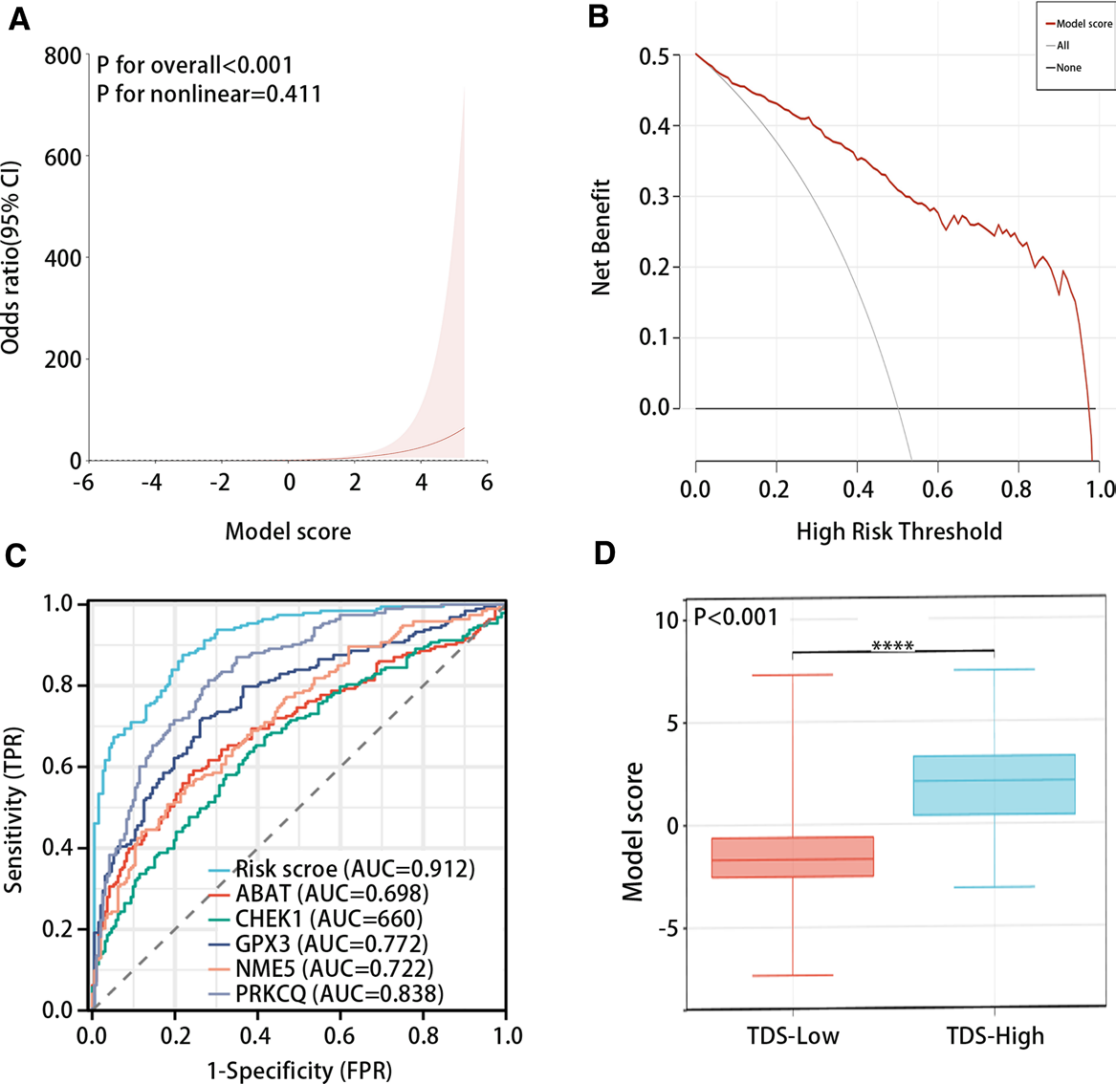
Diagnostic efficiency of the diagnostic model. (A) RSC analysis of TDS and diagnostic model. (B and C) DCA and ROC analysis of diagnostic model. (D) The relationship between model scores and TDS group. CI = confidence interval, DCA = decision curve analysis, FPR = false positive rate, RCS = restricted cubic spline, ROC = receiver operating characteristic, TPR = true positive rate.

**Figure 6.**
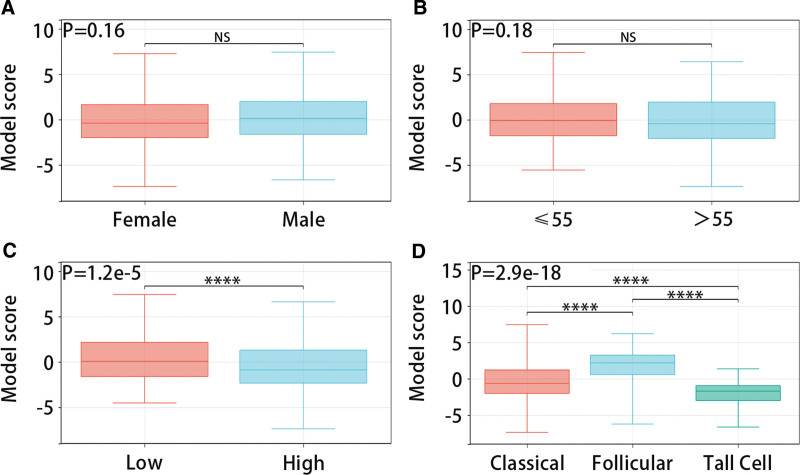
Relationship between diagnostic model and clinical characteristics. (A–D) The relationship between the diagnostic model and sex, age, pathological stage, and histological type.

The RCS analysis results presented that the diagnostic model had a linear relationship with high and low TDS groups (*P* for overall < .001, *P* for nonlinear = .411) (Fig. 5A). The DCA and ROC analysis results indicated that the model had great predictive efficiency for high and low TDS in PTC patients (Fig. 5B and C). Figure 5D evidenced that the model score was low in the TDS low group and high in the TDS high group (*P* < .05). Figure 6 presented that the diagnostic model was not statistically significant with gender and age, but it was significantly related to pathological stage (Low: stage Ⅰ and stage Ⅱ; High: stage Ⅲ and stage Ⅳ) and histological type.

**Figure 7.**
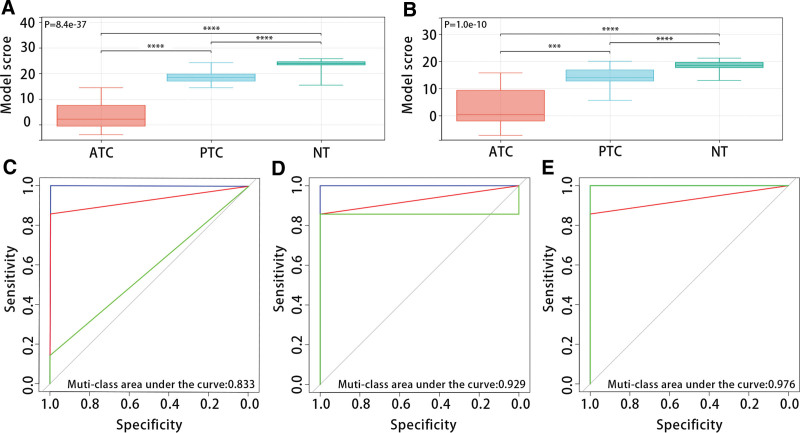
Validation of diagnostic model in GEO data set. (A-B) Expression of model scores in ATC, PTC, and NT based on GSE33630 and GSE29265 data sets. (C-D) In the GSE33630 data set, multi-classification ROC analysis of model score predicts the effectiveness of ATC, PTC, and NT respectively. ATC = anaplastic thyroid carcinoma, GEO = gene expression omnibus, NT = normal thyroid, PTC = papillary thyroid carcinoma, ROC = receiver operating characteristic.

Figure 7A and B exhibited that the diagnostic model was closely related to THCA patient types, and the model scores of ATC patients, PTC patients, and NT patients in the GSE33630 and GSE29265 datasets increased in turn (*P* < .05). The multi-class ROC analysis results displayed that the model had good predictive efficiency for patient types in the GSE33630 dataset (ATC, muti-class area under the curve:0.833; PTC, muti-class area under the curve:0.929; NT, muti-class area under the curve:0.979) (Fig. 7C–E).

**Figure 8.**
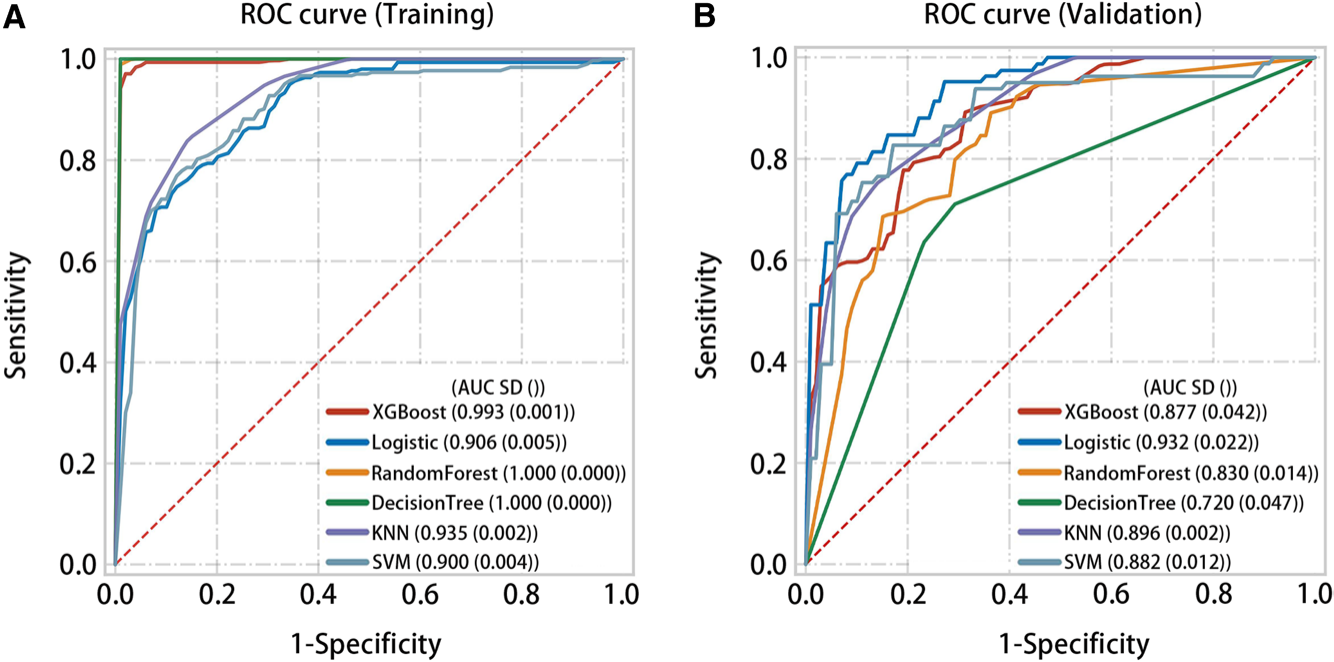
A variety of machine learning algorithms validate the predictive effectiveness of the diagnostic score model on both normal and THCA samples. (A) In the training set, the diagnostic score predicted the normal and THCA samples well. (B) In the validation set, the diagnostic score predicted the normal and THCA samples well. THCA = thyroid cancer.

In the training set, XGBoost (AUC = 0.993, *P* < .05), logistic (AUC = 0.906, *P* < .05), RandomForest (AUC = 1.000, *P* < .05), DecisionTree (AUC = 1.0, *P* < .05), KNN (AUC = 0.935, *P* < .05), and SVM (AUC = 0.900, *P* < .05) predicted high accuracy of normal samples and THCA samples (Fig. 8A). In the validation set, multiple machine learning algorithms also had good predictive performance for normal samples and THCA samples (Fig. 8B).

### 3.5. Prognostic value of diagnostic model in patients with THCA

**Figure 9.**
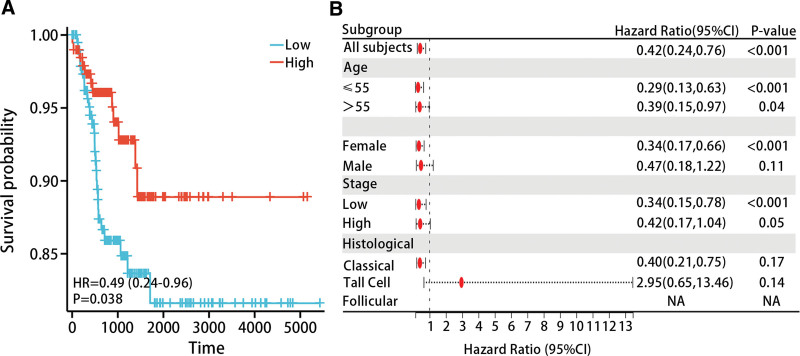
Low score model is related to poor prognosis. (A) Kaplan–Meier curve analysis shows that the low score model is related to the poor prognosis of THCA patients. (B) The relationship between the model score and the prognosis of patients with THCA in each clinical subgroup. HR = hazard ratio, THCA = thyroid cancer.

We performed K-M analysis to explore the relationship between the diagnostic model and prognosis of THCA patients. The results evidenced that lower model scores were associated with poorer progression free interval (PFI) in THCA patients (*P* < .05) (Fig. 9A). The results of subgroup analysis indicated that low model scores were associated with poorer PFI in THCA patients in the age ≤ 55 (HR = 0.42 (0.24–0.76), *P* < .05), age > 55 (HR = 0.39 (0.15–0.97), *P* < .05), female (HR = 0.34 (0.17–0.66), *P* < .05), and low stage (HR = 0.34 (0.15–0.78), *P* < .05) (Fig. 9B).

The study included age, gender, pathological stage, diagnostic model, and histological type in both univariate and multivariate Cox regression analysis. Univariate Cox regression showed that age, pathological stage, and diagnostic model were prognostic factors for THCA patients; multivariate Cox regression analysis revealed that pathological stage and diagnostic model could serve as independent prognostic factors for THCA patients (Table 4).

**Table 4:** Correlation between risk model and clinical characteristics and prognosis of patients with THCA.

Characteristic	Total (N)	Univariate analysis		Multivariate analysis	
		Hazard ratio (95% CI)	*P* value	Hazard ratio (95% CI)	*P* value
Histological type	489				
Classical	354	Reference			
Follicular	99	0.479 (0.213–1.074)	.074		
Tall Cell	36	0.294 (0.099–0.874)	.028		
Sex	489	0.600 (0.337–1.065)	.081		
Age	489	2.433 (1.403–4.218)	.002	1.727 (0.928–3.211)	.085
Pathological stage	489	2.694 (1.551–4.681)	<.001	1.956 (1.044–3.663)	.036
Model score group	489	0.474 (0.262–0.856)	.013	0.535 (0.294–0.971)	.040

CI = confidence interval, N = number, THCA = thyroid cancer.

### 3.6. Immune infiltration

**Figure 10.**
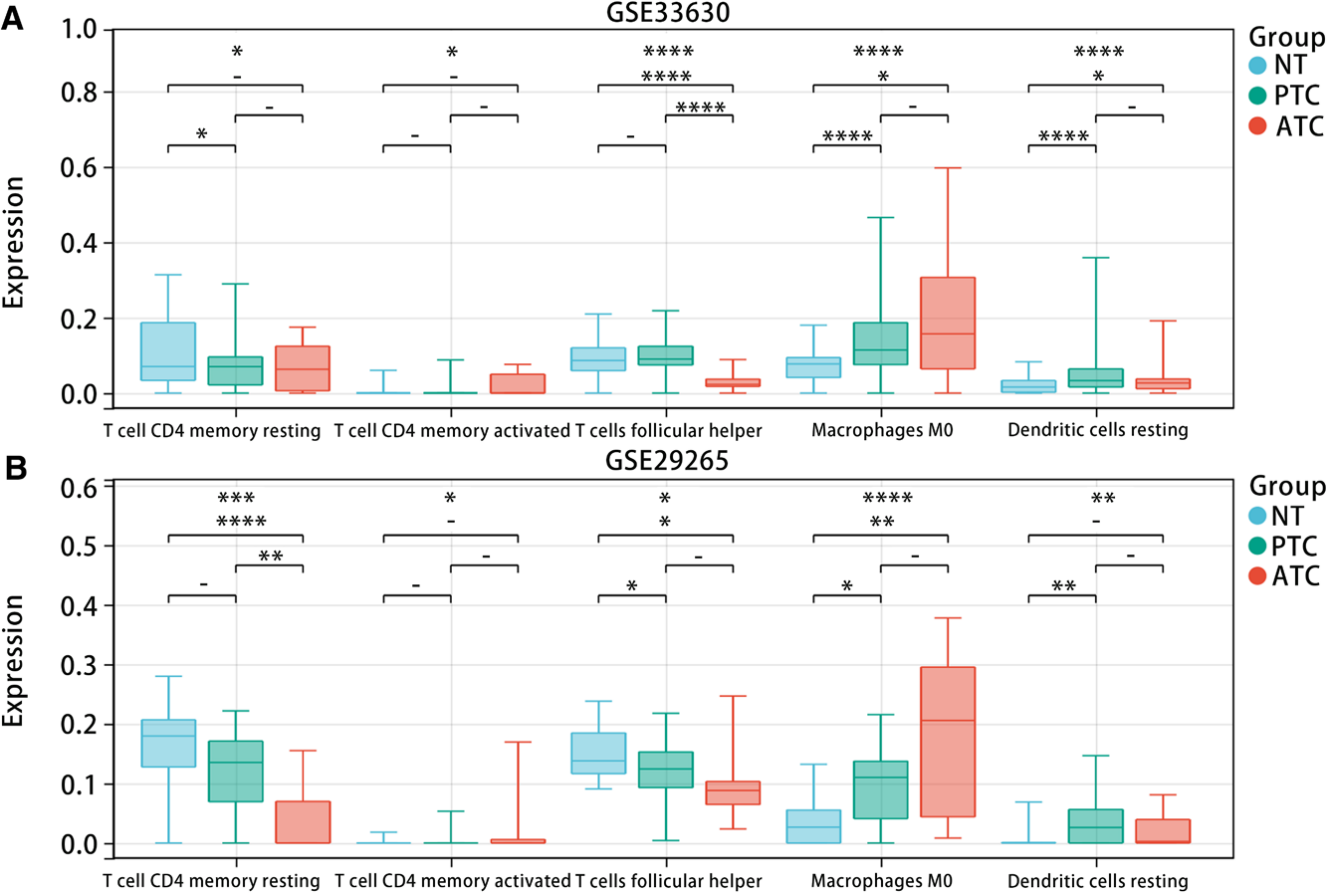
Relationship between infiltration level of immune cells and diagnostic model. (A and B) Immune cells differentially expressed in GSE33630 and GSE29265 data sets.

**Figure 11.**
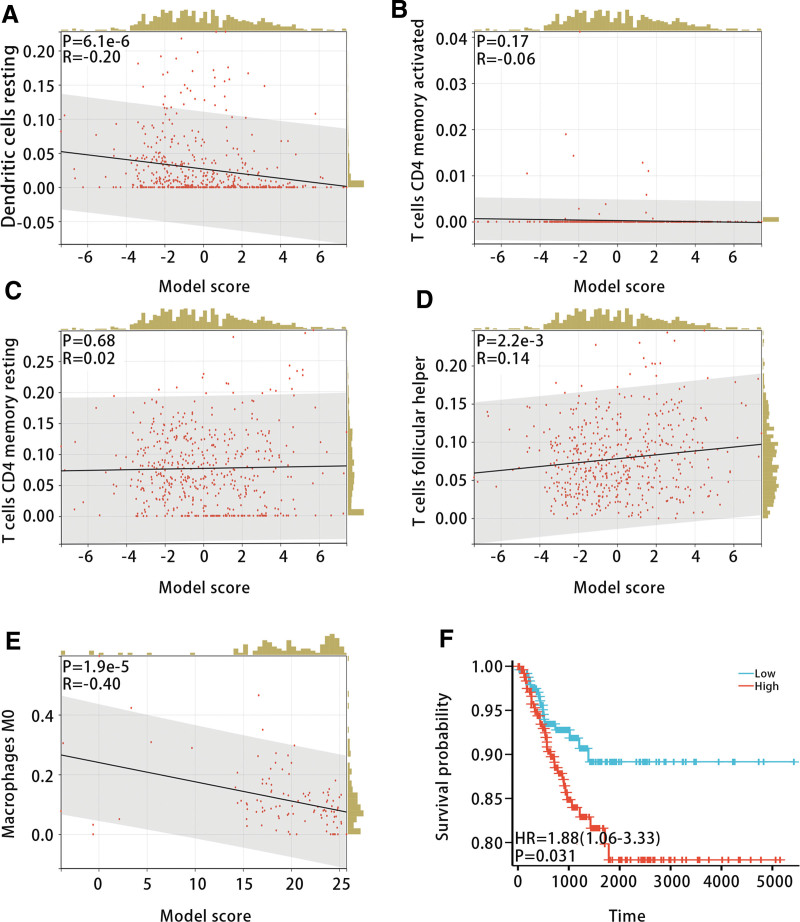
Relationship between immune cells and diagnostic model and PFI in patients with THCA. (A–E) The correlation between five kinds of immune cells (Dendritic cell resting, T cell CD4 memory activated, T cell CD4 memory resting, T cell follicular helper, and Macrophages M0) and the diagnostic model. (F) The high infiltration level of Macrophages M0 is related to the poor prognosis of THCA patients. Cor = correlation, PFI = progression-free interval, THCA = thyroid cancer.

To investigate the relationship between the diagnostic model and immune cell infiltration, we calculated the infiltration levels of 22 types of immune cells in samples from the GSE33630 and GSE29265 datasets using CIBERSORT. The results exhibited that there were differences in the infiltration levels of T cell CD4 memory resting, T cell CD4 memory activated, T cell follicular helper, Macrophages M0, and Dendritic cell resting among ATC patients, PTC patients and NT patients (Fig. 10A and B). In addition, we studied the relationship between the model score and the above immune cell infiltration levels. The correlation analysis displayed that the model score was most strongly and negatively correlated with Macrophages M0 (Fig. 11A–E). Furthermore, K-M analysis indicated that high infiltration levels of Macrophages M0 were associated with poorer PFI in THCA patients (*P* < .05) (Fig. 11F).

**Figure 12.**
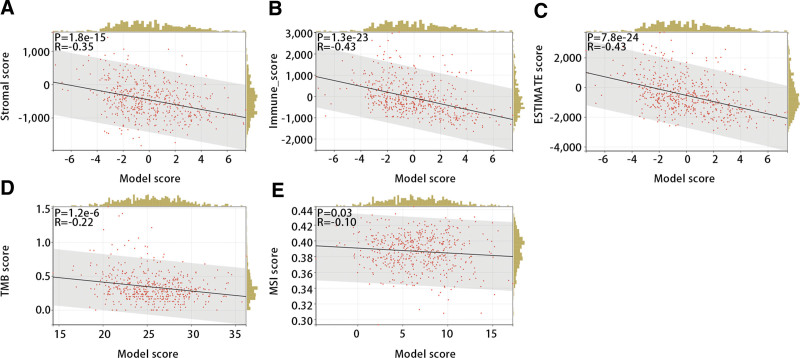
The relationship between the diagnostic model and (A) stromal score, (B) immune score, (C) ESTIMATE score, (D) TMB score, and (E) MSI score. MSI = microsatellite instability, TMB = tumor mutational burden.

Next, we evaluated the relationship of the diagnostic model with stromal score, immune score, ESTIMATE score, TMB score, and MSI score to further explore the function of the diagnostic model in immunotherapy. The Pearson correlation test displayed that all of them were significantly and negatively correlated with the diagnostic model (Fig. 12A–E) (*P* < .05).

### 3.7. Single-cell RNA-seq analysis

**Figure 13.**
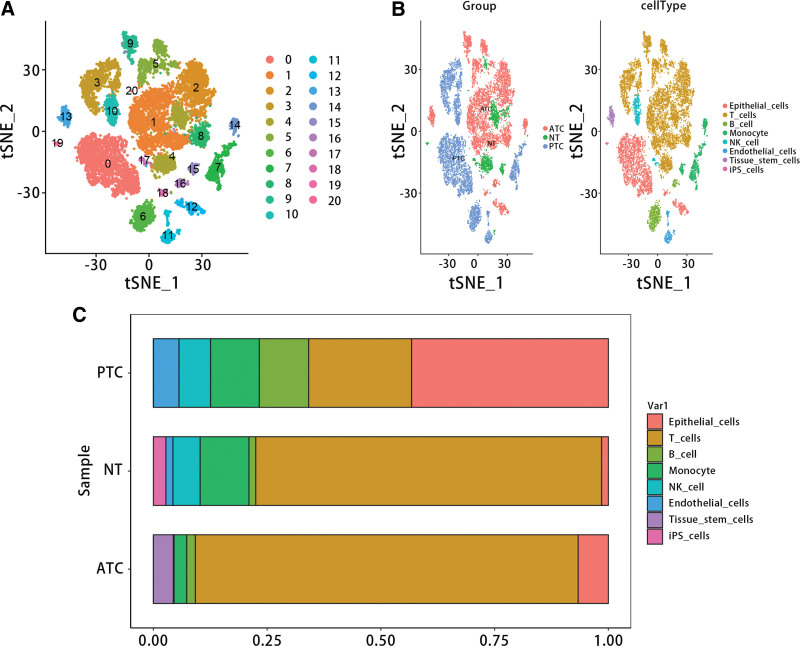
Single-cell RNA sequencing analysis. (A) t-Distributed Stochastic Neighbor Embedding (tSNE) clustering plot of all cells. (B) Cell distribution plot and annotated tSNE plot for each sample. (C) The proportion of major cell lines in each sample.

To further verify the expression of genes in different types of samples and the distribution of immune cells in different types of samples, we conducted a scRNA-seq data analysis. Figure 13A and B displayed that the scRNA-seq data were divided into 21 clusters, and these clusters were annotated as 8 cell types, including epithelial cells, T cells, B cells, monocyte, NK cells, endothelial cells, Tissue stem cells and iPS cells. Besides, it was obvious that the PTC had the highest proportion of epithelial cells, and ATC had highest proportion of T cells (Fig. 13C).

**Figure 14.**
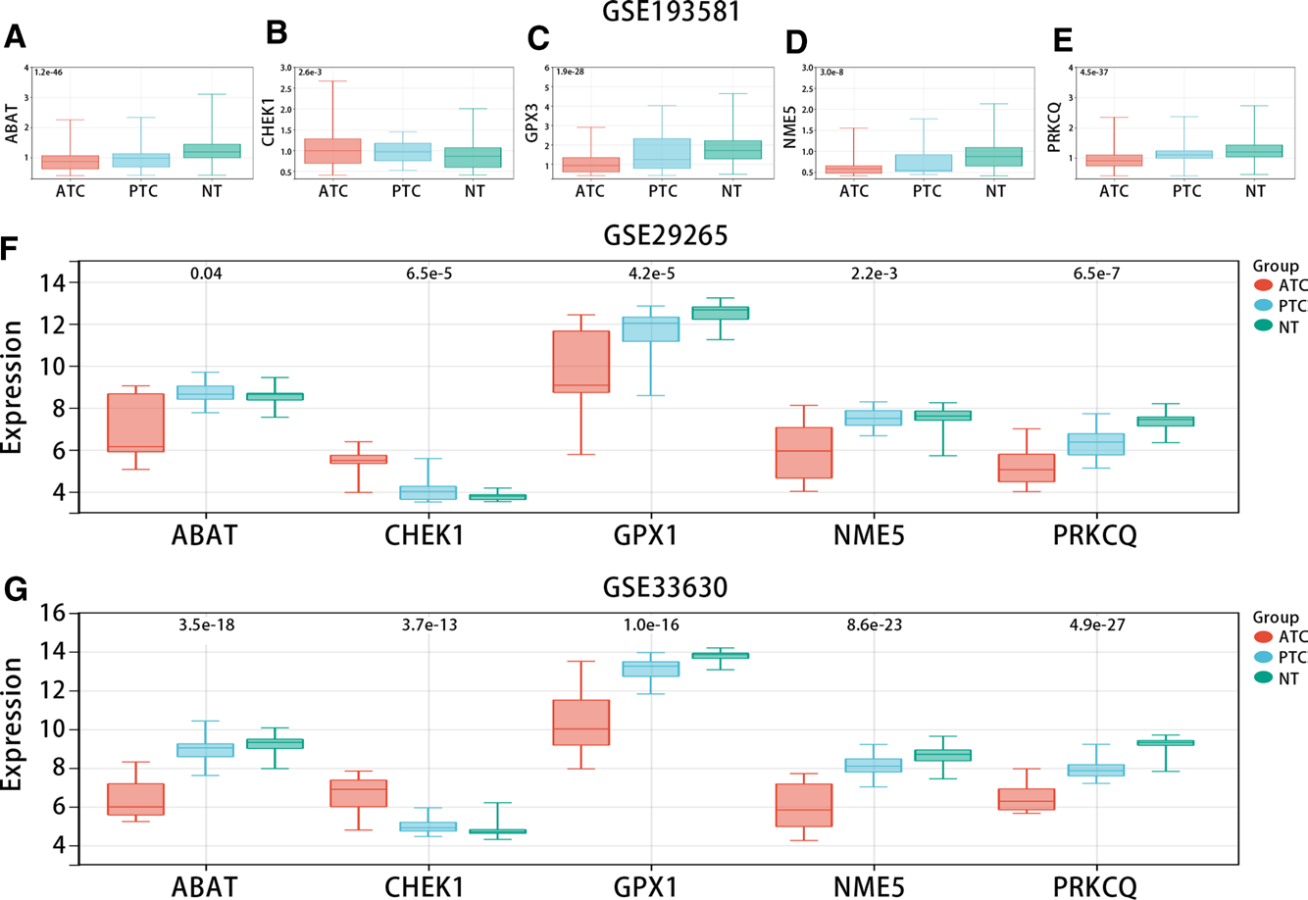
Expression of the genes in the model in GSE193581 (A), GSE29265 (B), and GSE33630 (C).

Figure 14A–E showed that CHEK1 was highly expressed in ATC compared with NT, but ABAT, GPX3, NME5, and PRKCQ were low expressed in the GSE193581 dataset. Consistent with scRNA results, in bulk RNA-seq data (GSE33630 and GSE29265), CHEK1 was highly expressed in ATC in comparison with NT, while ABAT, GPX3, NME5, and PRKCQ were low expressed (Fig. 14F and G).

### 3.8. Validation of gene expression in diagnostic model by qRT-PCR

**Figure 15.**
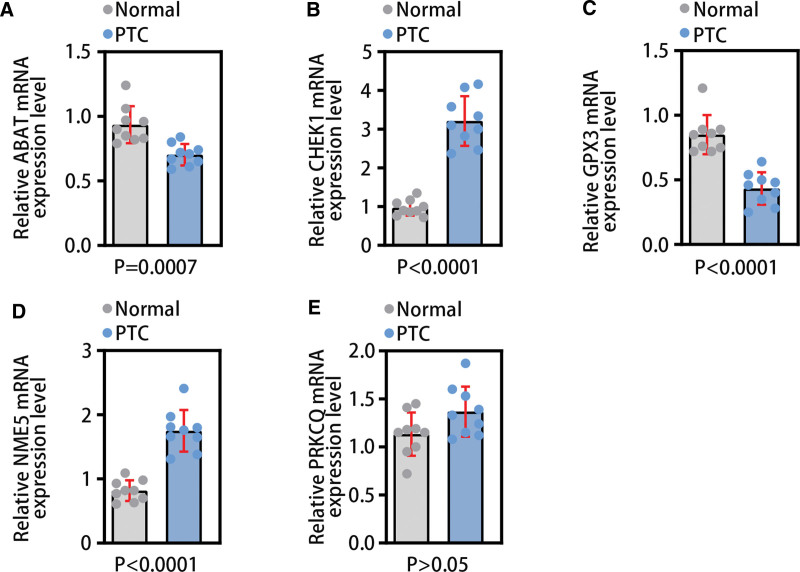
qRT-PCR validation. (A–E) The qRT-PCR results of ABAT, CHEK1, GPX3, NME5, and PRKCQ in normal tissues and PTC tissues, in sequence. PTC = papillary thyroid carcinoma.

To further verify the expression of genes in the diagnostic model in PTC tissues and normal tissues, we used qRT-PCR technology for validation. The results of qRT-PCR showed that ABAT (*P* < .001) and GPX3 (*P* < .001) were lower expressed in PCT tissues; CHEK1 (*P* < .001) and NME5 (*P* < .001) were highly expressed in PTC (Fig. 15A–E). The experimental results were generally consistent with the data analysis results of this study.

## 4. Discussion

Compared to differentiated THCA, advanced dedifferentiated THCA has an unsatisfactory response to chemotherapy and radiotherapy, and it is the main cause of death in THCA patients.^[20]^ DTC patients have a good prognosis after standard treatment (surgery, radioactive iodine therapy, and thyroid-stimulating hormone suppression therapy), but the diagnostic of local recurrence and distant metastasis can be as high as 20% to 10%. Two-thirds of the patients with local recurrence and distant metastasis have dysfunctional or even loss of sodium iodide symporter (NIS) expression in the basement membrane, ultimately leading to progressive loss of iodide uptake ability.^[21]^ Many tumors in PDTC and ATC could not concentrate iodine, making them unable to receive RAI treatment.^[22]^ Although methods for treating THCA continue to be improved and drugs are constantly innovating, there has been no significant improvement in patient survival. Therefore, we used TDS to construct a diagnostic model related to iodine metabolism and THCA subtypes, exploring its value in the classification, prognosis, and immunotherapy of THCA patients.

We first screened out 996 TDS-related genes and 104 intersection genes related to the THCA subtype. Secondly, LASSO analysis, correlation analysis, external dataset verification, and univariate and multivariate logistic regression analysis were used in turn to screen genes related to THCA subtype and TDS. Then, we used the results of multivariate logistic regression analysis to establish a diagnostic model with five genes.

The five genes in the diagnostic model that can independently predict TDS group included ABAT, CHEK1, GPX3, NME5, and PRKCQ. The experimental results also verified the expression level of these genes to some extent. ABAT (4-aminobutyrate aminotransferase) encodes GABAT. GABAT is closely related to the GABA transamination process and plays a role in the GABA shunt pathway.^[23]^ In clear cell renal cell carcinoma tissues, significant downregulation of ABAT promoted tumor growth, while overexpression of ABAT reduced cell proliferation and migration and impaired tumor metabolism in renal cancer cells.^[24]^ In liver cancer tissues, low expression of ABAT was associated with poor prognosis in hepatocellular carcinoma patients, while overexpression of ABAT could inhibit cancer cell behavior and suppress tumor formation in nude mice.^[25]^ In breast cancer patients, downregulated expression of ABAT was closely related to large tumor volume, high grade, metastatic tendency, poor prognosis, and chemotherapy resistance.^[26]^ CHEK1 (Checkpoint Kinase 1) is a central gene in the cell cycle and could regulate the cell cycle checkpoint to prevent DNA damage cells from undergoing mitosis and promote DNA repair. CHEK1 could regulate key genes involved in cell cycle arrest and DNA repair in the cell cycle pathway and P53 pathway.^[27]^ In breast cancer tissues, high expression of CHEK1 was associated with poor prognosis.^[28]^ In THCA tissues, CHEK1 expression levels increased in PTC patients and PDTC patients.^[16]^ In ovarian cancer tissues, overexpression of CHEK1 was closely related to poor prognosis.^[27]^ In various cancers, the efficacy of chemotherapy and radiation therapy combined with CHEK1 inhibitors was improved.^[29–31]^ GPX3 (Glutathione Peroxidase 3) is the only member of the GPX family that is related to secretion and plays an important role in detoxifying hydrogen peroxide and other oxygen free radicals.^[32]^ Studies have shown that low expression of GPX3 was positively correlated with poor prognosis in human cancers and had high diagnostic efficiency in breast cancer, colorectal cancer, tongue squamous cell carcinoma, and THCA.^[33]^ In THCA tissues, low expression of GPX3 impaired the clearance of intracellular superoxide compounds, leading to poor prognosis in THCA patients and being associated with lymph node metastasis, advanced age, and poor recurrence-free survival.^[34]^ PRKCQ (Protein Kinase C theta) encodes a serine/threonine kinase. PRKCQ is a T-cell specific PKC subtype that plays a key regulatory role in nuclear receptor-mediated active suppression.^[35]^ PRKCQ played a role in both PTC and gastrointestinal stromal tumors.^[36]^ PRKCQ could affect multiple processes of cancer, including cell proliferation, migration, and invasion.^[37]^ In colorectal cancer tissues, miR-128-1-5p can inhibit cell proliferation by suppressing PRKCQ expression.^[38]^ NME5 (Non-metastatic 5), as a member of the NME family, could participate in promoting tumor development. High expression of NME5 was associated with poor prognosis in breast cancer patients, and it may play a role in inhibiting cancer.^[39]^ Some studies have shown that NME5 plays an anti-cancer role in bladder cancer.^[40]^ NME5 could also be referred to as a p53-induced apoptosis-beta inhibitor (IPIA-beta), which could prevent Bax cell death.^[41]^ In a word, the five genes related to iodine metabolism and THCA subtype in the diagnostic model were closely related to the growth, proliferation, migration, and treatment of cancer.

Through exploring the relationship between the diagnostic model and TDS, we found that there is a linear relationship between the diagnostic model and TDS, and the diagnostic model has good predictive performance for TDS. Therefore, we can infer that the diagnostic model was closely related to TDS. Clinical feature analysis indicated that the diagnostic model was significantly associated with pathological stage and histological type, but it was not significantly related to gender and age. In the GSE33630 and GSE29265 datasets, the scores of ATC, PTC, and NT models increased in turn. The multi-class ROC results of the GSE33630 dataset showed that the diagnostic model had good predictive performance for THCA subtyping. The results of multiple machine learning algorithms showed that diagnostic scores had good predictive efficiency for normal samples and THCA samples. Therefore, we can infer that the diagnostic model was closely related to THCA subtyping. Our comprehensive analysis of K-M survival curves, subgroup analysis, and univariate and multivariate Cox regression analysis indicated that the diagnostic model was closely related to the prognosis in THCA patients, and low model scores were related to the unfavorable prognosis. Based on the results of the above analysis, we concluded that the diagnostic model was closely related to THCA subtyping and prognosis in THCA patients.

We explored the differences in immune cell infiltration levels among patients with different THCA subtypes. By integrating the results of immune infiltration from GSE33630 and GSE29265 datasets, we further explored the relationship between the diagnostic model and Macrophages M0. Correlation analysis and K-M survival curve analysis demonstrated that Macrophages M0 was negatively correlated with the diagnostic model, and high infiltration levels were unfavorable for the prognosis of THCA patients. The analysis of scRNA-seq data showed that epithelial cells, T cells, B cells, and monocytes were included in the samples of ATC, PTC, and NT. T cells follicular helper (Tfh) are important players in normal immune responses and autoimmune diseases, providing help signals to B cells in the germinal centers (GC) through contact-dependent and independent mechanisms.^[42]^ Secondly, Tfh cells can not only promote the proliferation and survival of B cells but also promote the formation of plasma cells and memory B cells.^[43]^ However, dysregulated Tfh cells trigger an abnormal GC B and memory B cell response, which produces autoantibodies and leads to the formation of B cells. Studies related to human/mouse have shown that abnormal Tfh is not only associated with autoimmune diseases, but also a pathogenic factor of autoimmune diseases.^[44]^ Studies have shown that CD4 T cells can promote tumor regression in cancer through a variety of mechanisms, including IL-2 secretion, enhancement of tumor-specific CD8 T cell function, or direct elimination of cancer cells.^[45]^ According to our results, we inferred that the hub genes may be involved in the occurrence of THCA through regulating the activation of Tfh and CD4 T memory cells.

In addition, the diagnostic model was negatively correlated with the matrix score, immune score, and ESTIMATE score. Numerous studies have shown that the infiltration of immune cells and stromal cells was closely related to the prognosis and treatment of tumor patients.^[46]^ The research results indicated that the diagnostic model was significantly negatively correlated with TMB and MSI. TMB and MSI as genomic biomarkers can be used to identify patients who may benefit from immune checkpoint inhibitors.^[47]^ Therefore, we inferred that the diagnostic model was significantly related to immune cells and immunotherapy-related indicators, which provided new biomarkers for immunotherapy in THCA.

There are certain limitations in this study. First, the public data sets involved in this paper may have defects such as batch effect, sample heterogeneity and lack of clinical information. Second, although machine learning and qRT-PCR were used in this paper to verify the predictive performance and expression of genes in the diagnostic model, further experiments need to be designed to verify the function and mechanism of genes involved in the model in THCA. Third, this paper only focuses on a specific population, which does not reflect the global situation of thyroid patients. We should then further test the risk model in different patient populations to assess its generality and applicability across different populations and geographic regions.

## 5. Conclusion

In conclusion, the diagnostic model was related to THCA subtyping and iodine metabolism. It could diagnose TDS subgroups and predict THCA subtypes and PFI of THCA patients. Additionally, this model was closely related to Macrophages M0 and immunotherapy-related indicators.

## Author contributions

**Conceptualization:** Qiu-ying Zhang.

**Data curation:** Qiu-ying Zhang, Yan Wang, De Gao.

**Formal analysis:** Qiu-ying Zhang, Pan-pan Wang.

**Investigation:** Da-wei Huo, Yue Li.

**Methodology:** Qiang Zhang, Da-wei Huo, Yue Li, Hai-chao Yan.

**Supervision:** Qiu-ying Zhang, Hai-chao Yan.

**Writing – original draft:** Qiu-ying Zhang, Yan Wang, Qiang Zhang, Da-wei Huo, Yue Li, De Gao, Pan-pan Wang.

**Writing – review & editing:** Hai-chao Yan.
